# Homa-adiponectin index as useful surrogate marker in the screeening of insulin resistance

**DOI:** 10.1186/1758-5996-7-S1-A135

**Published:** 2015-11-11

**Authors:** Brunna Sullara Vilela, Ana Carolina Junqueira Vasques, Roberta Soares Lara Cassani, Adriana Costa e Forti, José Carlos Pareja, Marcos Antônio Tambascia;, Investigators BRAMS, Bruno Geloneze

**Affiliations:** 1 Universidade Estadual de Campinas, Campinas, Brazil

## Background

The major adverse consequences of obesity are associated with the development of insulin resistance (IR) and adiposopathy. The Homeostasis Model Assessment-Adiponectin (HOMA-AD) was proposed as a modified version of the HOMA-IR, which incorporates adiponectin in the denominator of the index.

## Objectives

Evaluate the performance of the HOMA-AD compared with the HOMA-IR as a surrogate marker of IR in women, and to establish the cutoff value of the HOMA-AD.

## Materials and methods

The BRAMS is a cross-sectional multicenter survey. The data from 1.062 subjects met the desired criteria: 18-65 yrs. old, BMI: 18.5-49.9 Kg/m^2^ and non-diabetic. The IR was assessed by the indexes HOMA-IR and HOMA-AD (total sample) and by the hyperglycemic clamp (n=49). Metabolic syndrome was defined using the IDF criteria.

## Results

For the IR assessed by the clamp, the HOMA-AD demonstrated a stronger coefficient of correlation (r=-0.64) compared with the HOMA-IR (r=-0.56); p<0.0001. In the ROC analysis, compared with the HOMA-IR, the HOMA-AD showed higher values of the AUC for the identification of IR based on the clamp test (AUC: 0.844 vs. AUC: 0.804) and on the metabolic syndrome (AUC: 0.703 vs. AUC: 0.689), respectively; p<0.001 for all. However, the pairwise comparison did not suggest superiority for the HOMA-AD in the diagnostic of IR (p>0.05). The optimal cutoff identified for the HOMA-AD for the diagnosis of IR was 0.51.

## Conclusions

HOMA-AD was demonstrated to be a useful surrogate marker for detecting IR among adult women and presented a similar performance as the HOMA-IR.

